# Redefining nitric oxide production in legume nodules through complementary insights from electron paramagnetic resonance spectroscopy and specific fluorescent probes

**DOI:** 10.1093/jxb/ery159

**Published:** 2018-04-26

**Authors:** Laura Calvo-Begueria, Maria C Rubio, Jesús I Martínez, Carmen Pérez-Rontomé, Maria J Delgado, Eulogio J Bedmar, Manuel Becana

**Affiliations:** 1 Departamento de Nutrición Vegetal, Estación Experimental de Aula Dei, Consejo Superior de Investigaciones Científicas (CSIC), Apartado, Zaragoza, Spain; 2 Instituto de Ciencia de Materiales de Aragón, Universidad de Zaragoza-CSIC, Pedro Cerbuna, Zaragoza, Spain; 3 Departamento de Microbiología y Sistemas Simbióticos, Estación Experimental del Zaidín (CSIC), Profesor Albareda, Granada, Spain

**Keywords:** Denitrification, electron paramagnetic resonance, leghemoglobin, nitric oxide, nitrogen fixation, symbiosis

## Abstract

Nitric oxide (NO) is a signaling molecule with multiple functions in plants. Given its critical importance and reactivity as a gaseous free radical, we have examined NO production in legume nodules using electron paramagnetic resonance (EPR) spectroscopy and the specific fluorescent dye 4,5-diaminofluorescein diacetate. Also, in this context, we critically assess previous and current views of NO production and detection in nodules. EPR of intact nodules revealed that nitrosyl-leghemoglobin (Lb^2+^NO) was absent from bean or soybean nodules regardless of nitrate supply, but accumulated in soybean nodules treated with nitrate that were defective in nitrite or nitric oxide reductases or that were exposed to ambient temperature. Consequently, bacteroids are a major source of NO, denitrification enzymes are required for NO homeostasis, and Lb^2+^NO is not responsible for the inhibition of nitrogen fixation by nitrate. Further, we noted that Lb^2+^NO is artifactually generated in nodule extracts or in intact nodules not analyzed immediately after detachment. The fluorescent probe detected NO formation in bean and soybean nodule infected cells and in soybean nodule parenchyma. The NO signal was slightly decreased by inhibitors of nitrate reductase but not by those of nitric oxide synthase, which could indicate a minor contribution of plant nitrate reductase and supports the existence of nitrate- and arginine-independent pathways for NO production. Together, our data indicate that EPR and fluorometric methods are complementary to draw reliable conclusions about NO production in plants.

## Teaser

The combined use of EPR spectroscopy and the specific fluorescent dye 4,5-diaminofluorescein diacetate uncovers sites of nitric oxide production in legume nodules and warns of potential artifacts.

## Introduction

Nitric oxide (NO) is a gaseous free radical and signal molecule involved in a vast array of physiological processes of plants, including legume nodule formation and development (Hichri *et al.*, 2016). Thus, NO was detected after infection of roots by rhizobia in the model legumes *Lotus japonicus* (Nagata *et al.*, 2008) and *Medicago truncatula* (del Giudice *et al.*, 2011). In *L. japonicus* roots inoculated with *Mesorhizobium loti*, the NO concentration increases after ~4 h and then decreases due to the induction of a non-symbiotic hemoglobin (LjGlb1-1) which scavenges NO and thus avoids triggering the defense response of the plant (Nagata *et al.*, 2008; Fukudome *et al.*, 2016). Mutant plants of *L. japonicus* defective in LjGlb1-1 have lower infection rates, fewer nodules, and a higher NO level in roots than the wild-type (WT) plants, indicating that this hemoglobin is required for *M. loti* infection, probably by regulating the NO level in the roots (Fukudome *et al.*, 2016). Likewise, several studies with NO scavengers and NO biosensor bacterial strains have shown that NO production is critical at the early stages of the *M. truncatula*–*Sinorhizobium meliloti* interaction (del Giudice *et al.*, 2011).

The homeostasis of NO is also important in mature and senescent nodules because of the dual effects of NO. On the one hand, NO inhibits nitrogenase activity (Trinchant and Rigaud, 1982; Sasakura *et al.*, 2006; Kato *et al.*, 2010) and is the precursor of nitrating molecules that can alter the activity of key nodule proteins such as glutamine synthetase and leghemoglobin (Lb) through tyrosine nitration (Melo *et al.*, 2011; Sainz *et al.*, 2015) or heme nitration (Navascués *et al.*, 2012). On the other hand, low and steady NO concentrations are needed to maintain nodule functioning (Shimoda *et al.*, 2005; Cam *et al.*, 2012). The major sources of NO in nodules are the cytosolic nitrate reductase (NR) and the mitochondrial electron transport chain in the host cells, and the periplasmic nitrate reductase (Nap) and the respiratory nitrite reductase (NirK) in the bacteroids (Meakin *et al.*, 2007; Sánchez *et al.*, 2010; Horchani *et al.*, 2011). Additional possible sources of NO, such as a putative NO synthase (NOS) activity initially reported in lupine nodules (Cueto *et al.*, 1996), remain to be identified.

Several studies have examined NO production in nodules. EPR and Soret–visible spectroscopies were used to detect the highly stable nitrosyl-leghemoglobin (Lb^2+^NO) complex in crude Lb preparations, nodule extracts, or intact nodules (Maskall *et al.*, 1977; Kanayama *et al.*, 1990; Mathieu *et al.*, 1998; Meakin *et al.*, 2007; Sánchez *et al.*, 2010). On the other hand, using a specific dye, NO was found in nodules of *M. truncatula* and alfalfa but not in those of peanut (Baudouin *et al.*, 2006; Maiti *et al.*, 2012; Meilhoc *et al.*, 2013). Because of the equivocal nature of these results, we have undertaken a detailed study to detect, localize, and compare NO production by using EPR in intact nodules and a fluorescent dye in nodule sections. To this end, we chose soybean and bean for two reasons: (i) these legumes produce nodules with a well-defined determinate growth pattern (Minchin *et al.*, 2008) in which NO has not been localized to date; and (ii) a comparison of NO production in bean and soybean nodules is useful to gain insight into the contribution of NO_3_^−^ as an NO precursor because soybean nodule bacteroids express Nap and other enzymes of the denitrification pathway (Sánchez *et al.*, 2010), whereas bean nodule bacteroids are devoid of respiratory nitrate and nitrite reductases (Becana *et al.*, 1989). Here, we used both legumes, along with bradyrhizobial mutants defective in denitrification enzymes, to examine NO production in the absence and presence of NO_3_^−^. Based on our findings, we suggest that only EPR of intact nodules that have been flash-frozen and analyzed immediately after their detachment provides genuine measurements of NO production *in vivo*, albeit the method is limited to NO generated in the infected zone. Notably, NO was undetectable by EPR in bean or soybean nodules regardless of NO_3_^−^ supply, but it was observed in soybean nodules treated with NO_3_^−^ that lack bacteroid NirK or nitric oxide reductase (Nor), or that were left at 23 °C for 1 h. Our results also indicate that fluorescent dyes cannot be used to quantify NO production but only to assess the potential of nodule cells to generate NO. This technique allowed us to localize NO in the infected cells of the central zone, but also in the mid/inner cortex (nodule parenchyma), where the O_2_ diffusion barrier (ODB) is located. In the light of these and other data described in detail here, we critically discuss previous and current views of NO production and detection in legume nodules.

## Materials and methods

### Biological material and plant growth

Common bean (*Phaseolus vulgaris* L. cv. Contender) seedlings were inoculated with *Rhizobium leguminosarum* bv. *phaseoli* strain 3622. Soybean (*Glycine max* Merr. cv. Williams) seedlings were inoculated with *Bradyrhizobium diazoefficiens* strain USDA110 or the mutant derivatives GRPA1 (*napA*), GRK308 (*nirK*), and GRC131 (*norC*), which are defective, respectively, in the enzymes Nap, NirK, and Nor of the denitrification pathway (Sánchez *et al.*, 2010). Both legumes were inoculated 7 d after germination and were grown in pots containing a perlite:vermiculite (1:1, v/v) mixture in a controlled-environment chamber, with a 24 °C/21 °C day/night regime, 16 h photoperiod, and 350 μmol m^−2^ s^−1^ light intensity. Plants were grown for ~30 d (bean) and ~35 d (soybean) in a nutrient solution omitting NO_3_^−^ (Matamoros *et al.*, 2006). For studies of NO_3_^−^-induced nodule senescence, plants were treated with 10 mM KNO_3_ in the nutrient solution for 4 d for bean and 3 d or 6 d for soybean. These plants were harvested at the same age as those not receiving KNO_3_.

### Leghemoglobin derivatives

Soybean Lb*a* and bean Lb*a* were purified in the ferric state (Lb^3+^) according to published protocols (Becana and Klucas, 1990). The ferrous form (Lb^2+^) was obtained by adding a trace of sodium dithionite and the oxyferrous form (Lb^2+^O_2_) by passing Lb^2+^ through a Sephadex G-25 mini-column (NAP-5; GE Healthcare). Lb^2+^NO was produced by incubating Lb^2+^ with the NO donor diethylamine NONOate (DEA; Sigma-Aldrich) as follows. To an Eppendorf tube containing 10 μl of 1.5 mM Lb^3+^ and 220 μl of 50 mM potassium phosphate buffer (pH 7.0), a trace of dithionite was added and gently dissolved by inversion to generate Lb^2+^. This solution was immediately mixed with 10 μl of 6 mM DEA and incubated at 23 °C for 5 min. Alternatively, Lb^2+^NO was generated by adding a trace of NO_2_^−^ to the Lb^2+^ solution.

For EPR measurements, the Lb^3+^, Lb^2+^, Lb^2+^O_2_, and Lb^2+^NO solutions were made up to 20% of glycerol inmediately after preparation. Glycerol avoids formation of microcrystalline ice that causes broadening and deformation of the EPR signal and can even break the EPR sample tubes. About 150 μl was loaded in the EPR tube. Samples were frozen by introducing the tube into the EPR cryostat and analyzed using conditions described below for bean and soybean nodules. All Lb forms were analyzed at a final concentration of 50 μM and their identities were confirmed by the Soret–visible spectra. All measurements were performed in two independent Lb preparations with identical results.

### NO detection with EPR

Nodules of similar size were harvested from plants and immediately introduced into cylindrical EPR tubes (3 mm internal diameter) under liquid nitrogen. The tubes were filled with nodules of a similar size (<20% variability), closely packed to a depth of ~3 cm, and were placed into an Oxford CF900 cryostat (Oxford Instruments, Eynsham, UK), and refrigerated by a continuous flow of liquid He, in the interior of the EPR cavity. The EPR spectrometer was a Bruker ELEXYS E580 (Bruker; Karlsruhe, Germany) operating at the X band (microwave frequency ~9.5 GHz). Typical measurement conditions were: temperature, 80 K; microwave power, 2 mW; modulation amplitude, 0.2 mT. The microwave power and modulation amplitude were chosen so that there was no signal saturation or distortion. The measured spectra were numerically smoothed by using an ‘adjacent-averaging’ filter in order to reduce noise without loss of signal. All EPR measurements were made in nodules from two series of bean and soybean plants grown independently with similar results. For EPR measurements of nodule extracts, 100 mg of bean and soybean nodules treated with NO_3_^−^ were homogeneized with 500 μl of 50 mM potassium phosphate buffer (pH 7.0) at 0 °C. The extracts were cleared by centrifugation (15000 *g*, 4 °C) and the soluble fraction was made to 20% with glycerol, frozen, and immediately analyzed.

### NO detection with a fluorescent probe

Fresh nodules were cut into 90 μm sections in 10 mM Tris–HCl (pH 7.4) and 10 mM KCl using a VT1000S vibratome (Leica; Wetzler, Germany). Sections were incubated for 30 min at 23 °C with 10 μM 4,5-diaminofluorescein diacetate (DAF-2 DA; Sigma-Aldrich), washed three times for 5 min with the same buffer, mounted on a slide with buffer:glycerol (1:1), and observed using a Leica SP2 confocal microscope with excitation at 488 nm and emission at 498–549 nm. To ascertain that NO was the reactive nitrogen species being produced, nodule sections were incubated with the NO scavenger 2-(4-carboxyphenyl)-4,4,5,5-tetramethylimidazoline-1-oxyl-3-oxide (cPTIO; Calbiochem). This compound was used at a concentration of 1 mM for 1 h in the dark and was also added to the incubation medium with DAF-2 DA.

For studies with enzyme inhibitors, nodule sections were incubated in the dark with 1 mM or 5 mM *N*^G^-monomethyl-l-arginine (l-NMMA), *N*^G^-nitro-l-arginine (l-NNA), or sodium tungstate (Na_2_WO_4_) for 1–2 h at 23 °C. Arginine analogs were purchased from Calbiochem (La Jolla, CA, USA) and Na_2_WO_4_ from Sigma-Aldrich. After removing the solution, the nodule sections were incubated for 30 min with 10 μM DAF-2 DA along with the corresponding freshly prepared inhibitors. The sections were then washed twice, mounted on slides, and visualized by confocal laser scanning microscopy (CLSM) as indicated above.

For studies of co-localization of NO production and bacteroids, nodule sections were incubated with 10 μM DAF-2 DA and 1 μM SYTO 83 (Life Technologies, USA) for 30 min in the dark. The sections were then washed twice, mounted, and observed by CLSM with the same settings as above for DAF-2 DA and with excitation at 543 nm and emission at 560–615 nm for SYTO 83.

All CLSM studies were performed using nodules from at least two series of bean and soybean plants grown independently with similar results, and representative images are shown in all the figures.

## Results and Discussion

### Identification of EPR signals of leghemoglobins *in vitro* and *in vivo*

**Fig. 1.**
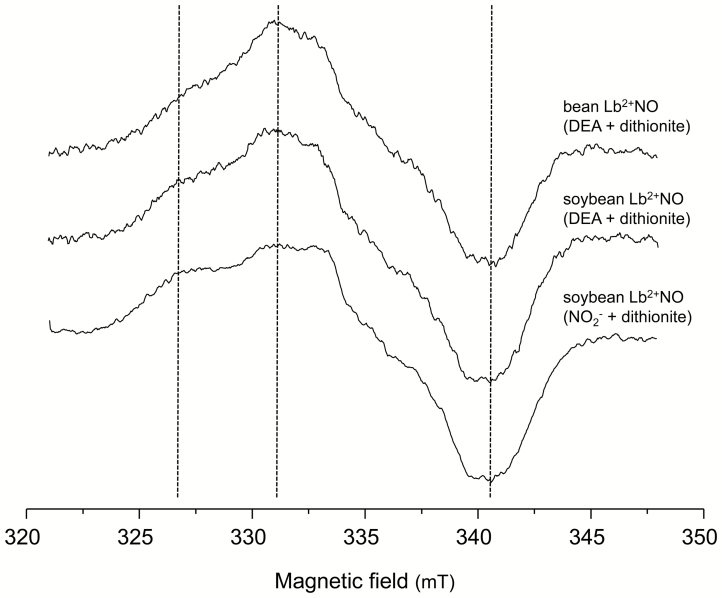
EPR spectra of the Lb^2+^NO complex obtained by two methods. Vertical dotted lines indicate the main features of the Lb^2+^NO signal, with a shoulder at 326 mT, a maximum at 331 mT, and a minimum at 341 mT.

Under physiological conditions, soybean nodules contain ~80% Lb^2+^, ~20% Lb^2+^O_2_, and, if any, negligible amounts of Lb^3+^ (Appleby, 1984; Lee *et al.*, 1995). Consequently, a prerequisite to attempt to detect Lb^2+^NO in nodules was to characterize the EPR spectra of all those Lb species using purified proteins. Supplementary Fig. S1 at *JXB* online shows that, under our measurement conditions, Lb^2+^ and Lb^2+^O_2_ were EPR silent, whereas Lb^2+^NO displayed a signal in the 320–350 mT (*g ~*2.0) region. More specifically, Fig. 1 shows that the Lb^2+^NO signal has a characteristic shape with an increasing shoulder at 326 mT, a maximum at 331 mT, and a sharp minimum at 341 mT. In this figure it can also be observed that identical spectra were obtained when Lb^2+^NO was produced from Lb^3+^ with an NO donor (DEA) plus dithionite or with NO_2_^−^ plus dithionite. We associated this signal with an electronic spin *S*=1/2 with principal values of the *g* tensor *g*_x_ ~2.07, *g*_y_ ~2.00, and *g*_z_ ~1.97, and a partially unresolved hyperfine interaction with one ^14^N nucleus; the signal is typical of the heme^2+^-NO species as previously reported for hemoglobin (Doetschman and Utterback, 1981) and cytochrome P-450 (Tsubaki *et al.*, 1987). Moreover, Lb^3+^ typically displayed a signal in the 100 mT (*g* ~6.0) region, but this signal was noticeable only at low temperatures (<25 K) and could not be detected at 80 K (Supplementary Fig. S2).

**Fig. 2.**
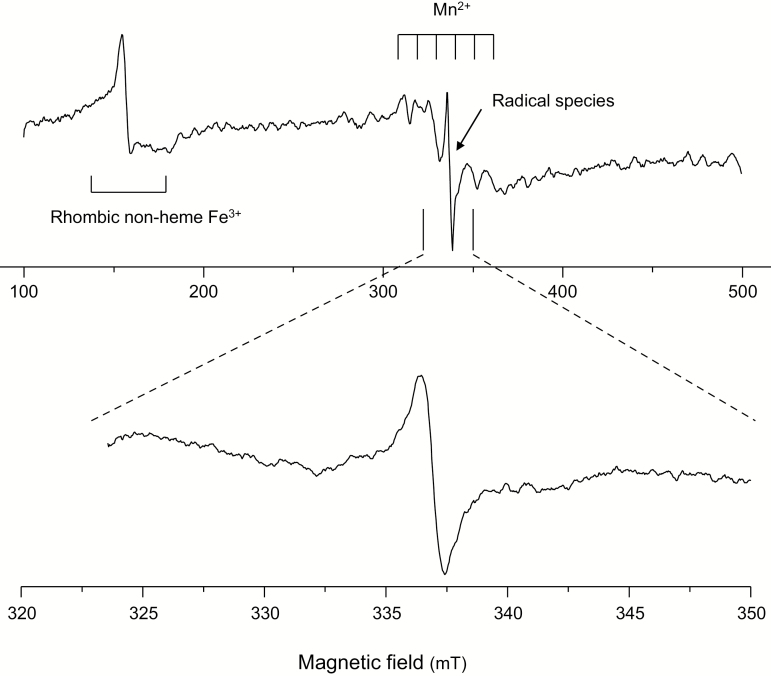
EPR spectrum of intact soybean nodules showing three types of features. These correspond to the signals of rhombic non-heme Fe^3+^ (*g*=4.3), Mn^2+^ (six equally spaced lines centered at *g*=2.0), and organic free radicals (intense narrow signal at *g*=2.0). The Lb^2+^NO signal, if present, appears superimposed on these features (see text for details).

The next necessary step of our study was to characterize the EPR signals of intact nodules. Figure 2 shows a typical EPR spectrum of intact soybean nodules, in which three main features can be distinguished: (i) a single, non-symmetric signal in the 150 mT region (*g* ~4.3) which is due to one or several non-heme Fe^3+^ species in a rhombic or low symmetry environment; (ii) a feature spreading out in the range of 300–360 mT which is characterized by six equally spaced signals and corresponds to one or several Mn^2+^ species; and (iii) a rather narrow, intense signal at 337 mT (*g* ~2.0) which can be ascribed to one or several organic radical species. Intact bean nodules showed similar spectral features (rhombic non-heme Fe^3+^, Mn^2+^, and radical species), albeit the pattern associated with Mn^2+^ was relatively more intense and the six-line pattern signal was more defined (Supplementary Fig. S3).

**Fig. 3.**
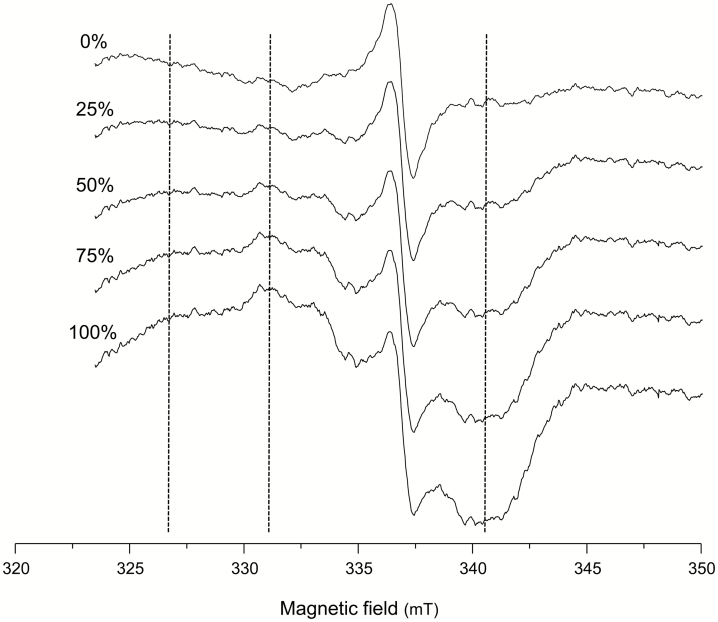
EPR spectra obtained by numerical addition of the signals of intact soybean nodules and different percentages of authentic Lb^2+^NO. Direct comparison of the experimental data with these spectra allows the demonstration of the presence of Lb^2+^NO. Vertical dotted lines indicate the main features of the Lb^2+^NO signal, with a shoulder at 326 mT, a maximum at 331 mT, and a minimum at 341 mT.

These three distinctive signals were found in all soybean and bean nodule samples examined in our study. The EPR signal of Lb^2+^NO, if present in nodules, would overlap with signals (ii) and (iii), as may be inferred from the spectral characteristics of purified Lb^2+^NO (compare the range of 320–350 mT in Figs 1 and 2). In this field range, the Mn^2+^ species displays a positive shoulder at 324 mT and a negative shoulder at 342 mT, whereas the signal of Lb^2+^NO is narrower than all the other features and is readily distinguished. To visualize this, we performed a numerical addition of the spectra of intact soybean nodules and the spectra of authentic Lb^2+^NO at variable proportions (Fig. 3). This figure predicts that nodules containing Lb^2+^NO will show spectra with a clear diagnostic signal in the range of 320–345 mT.

### Nitrosyl-leghemoglobin only occurs at significant concentrations in soybean nodules defective in bacteroid nitrite or nitric oxide reductases

**Fig. 4.**
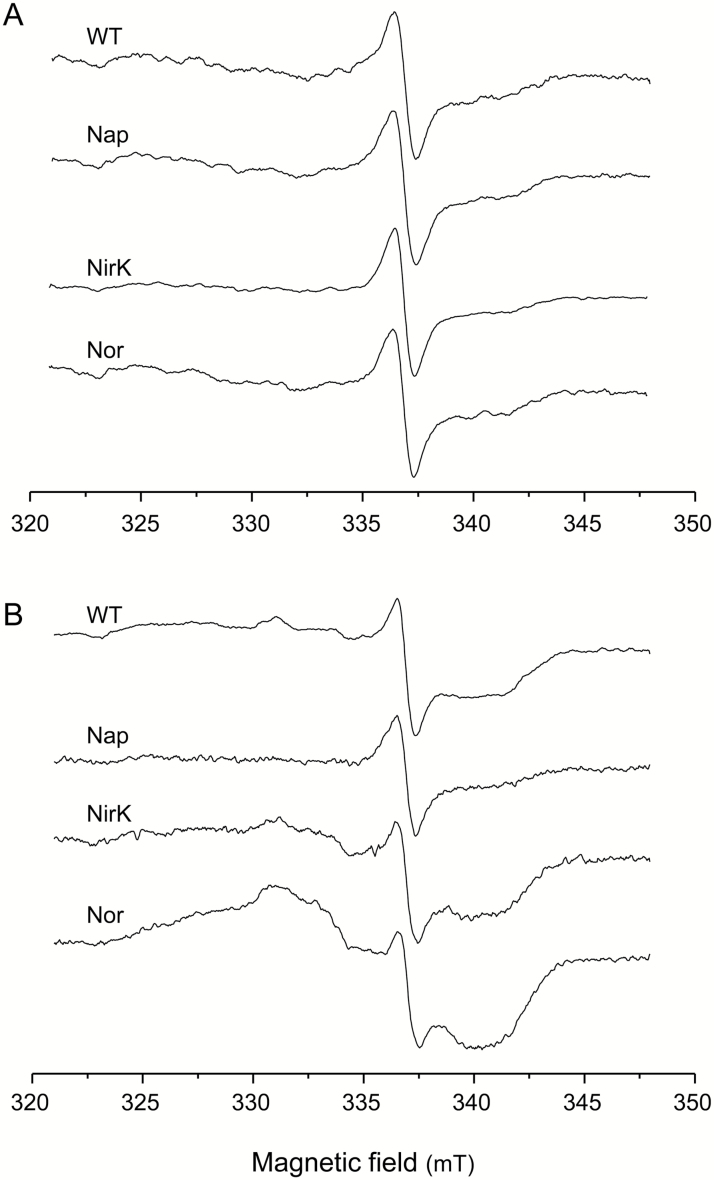
EPR spectra of intact soybean nodules. Plants nodulated with *B. diazoefficiens* strain USDA110 (WT) and the mutant derivatives *napA*, *nirK*, or *norC* were treated with 10 mM KNO_3_ for (A) 3 d or (B) 6 d.

The observations described so far indicate that EPR is an excellent method to identify Lb^2+^NO in nodules for two reasons: (i) the nitrosyl complex has diagnostic spectral features compared with other Lb forms; and (ii) EPR can be used with intact nodules, precluding possible artifacts that may arise during nodule sectioning or extraction. In this study, we performed experiments to detect Lb^2+^NO by EPR in bean and soybean nodules treated or not with NO_3_^−^. Soybean nodules were produced with the WT strain as well as with the bradyrhizobial mutants *napA*, *nirK*, and *norC*. To avoid artifacts, it was critical to collect intact nodules directly from the plants into the EPR tubes, while immersed in liquid nitrogen. Following this procedure, we were unable to detect Lb^2+^NO in bean nodules with or without NO_3_^−^ (Supplementary Fig. S4) or in soybean nodules formed by any of the strains after 3 d with NO_3_^−^ (Fig. 4A). In contrast, Lb^2+^NO was clearly observed in soybean nodules formed by the *nirK* or *norC* mutants after 6 d with NO_3_^−^ (Fig. 4B). The diagnostic signal of Lb^2+^NO appears to overlap with those of organic radicals, between 323 mT and 345 mT, exactly as in the predicted spectra of Fig. 3. Indeed, the comparison of spectra in Figs 3 and 4 enabled us to identify Lb^2+^NO in nodules. The spectra of the WT, NirK, and Nor nodules in Fig. 4B are similar, respectively, to the spectra containing 25, 50, and 75% Lb^2+^NO shown in Fig. 3. In contrast, the spectra of Fig. 4A, as well as the spectrum of Nap-deficient nodules of Fig. 4B, lack the Lb^2+^NO signal. Therefore, the Nap enzyme contributes to Lb^2+^NO production in nodules after 6 d with NO_3_^−^.

**Fig. 5.**
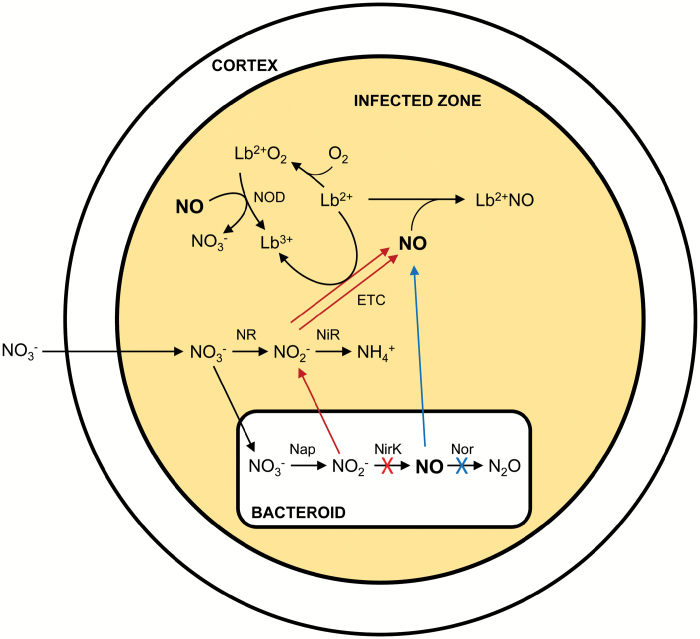
Scheme showing NO production in soybean nodules. The pathways for NO production in nodules formed by the *nirK* and *norC* mutants are marked in red and blue, respectively. Common reactions are in black. For simplicity, the last denitrification step (reduction of N_2_O to N_2_ by nitrous oxide reductase) of bacteroids is omitted. ETC, electron transport chain; NR, cytosolic nitrate reductase; NiR, plastidic nitrite reductase; NOD, NO dioxygenase.

The simpler explanation for our observations of Lb^2+^NO *in vivo* is that NO_3_^−^ has restricted access to the bacteroids after 3 d (Sprent *et al.*, 1987; Becana *et al.*, 1989) because otherwise nodules lacking NirK or Nor would contain Lb^2+^NO at this stage. An alternative explanation, however, is that NO_3_^−^ is present in the bacteroids after 3 d (Arrese-Igor *et al.*, 1998), but the generated NO is scavenged by metabolic reactions. Such scavenging may occur by nitrosylation of protein cysteine residues or, most probably, by the NO dioxygenase (NOD) activity of Lb^2+^O_2_ that converts NO into NO_3_^−^ in the cytosol of infected cells (Calvo-Begueria *et al.*, 2017). In this scenario, Lb^2+^NO would be detectable in nodules only when at least two mechanisms controlling NO concentration, NOD activity in the cytosol and NirK or Nor activities in the bacteroids, were overwhelmed (Fig. 5). Thus, after 6 d with NO_3_^−^, the *nirK* nodules accumulate NO_2_^−^, which diffuses out of the bacteroids and is reduced to NO by Lb^2+^. This Lb form is found at concentrations of 1–2 mM in nodules and may act as a dissimilatory nitrite reductase generating NO. This is shown *in vitro* by reducing soybean Lb^3+^ to Lb^2+^ with a trace of dithionite and then adding NO_2_^−^ (Fig. 1), and has been reported for other ferrous hemoglobins of plants and cyanobacteria (Sturms *et al.*, 2011). It cannot be entirely ruled out, however, that NO_2_^−^ is also reduced by the mitochondrial electron transport chain (Horchani *et al.*, 2011). In the *norC* nodules, NO accumulates, diffuses out of the bacteroids, and binds to cytosolic Lb^2+^. It is thus evident from our results that bacteroid NirK and Nor act sequentially to keep NO under control. This would prevent the accumulation of functionally inactive Lb^2+^NO, thereby protecting N_2_ fixation.

### Nitrosyl-leghemoglobin is artifactually generated in nodule extracts or in intact nodules soon after detachment

Our results showing that Lb^2+^NO is absent from WT nodules exposed to NO_3_^−^ for up to 6 d refute the proposal that Lb^2+^NO is responsible for the inhibition of N_2_ fixation by NO_3_^−^ and is also in sharp contrast to the detection of Lb^2+^NO by Soret–visible spectroscopy in extracts from soybean and cowpea nodules (Maskall *et al.*, 1977; Kanayama *et al.*, 1990; Kanayama and Yamamoto, 1991; Meakin *et al.*, 2007; Sánchez *et al.*, 2010). These contradictory results led us to investigate whether Lb^2+^NO was formed artifactually during extraction of nodule Lb. Supplementary Fig. S5 shows EPR spectra of soluble extracts from bean nodules treated with 10 mM KNO_3_ for 4 d and from soybean nodules treated similarly for 3 d or 6 d. The extracts were prepared at 0 °C, cleared by centrifugation, and frozen in liquid nitrogen. We could not detect Lb^2+^NO in bean nodule extracts, as occurred for the corresponding intact nodules (Supplementary Fig. S4), which may be explained by the absence of denitrifying enzymes in bean nodule bacteroids. However, Lb^2+^NO was found in both soybean nodule extracts at similarly high levels (Supplementary Fig. S5), which disagrees with the observations on the respective intact nodules (spectra of WT nodules in Fig. 4A and B) and unveils the artifactual origin of Lb^2+^NO. This artifact may occur when NO_3_^−^ in the cortex of soybean nodules, which may be at a relatively high concentration (Becana *et al.*, 1989; Arrese-Igor *et al.*, 1998), is brought into contact with the bacteroids during nodule extraction.

**Fig. 6.**
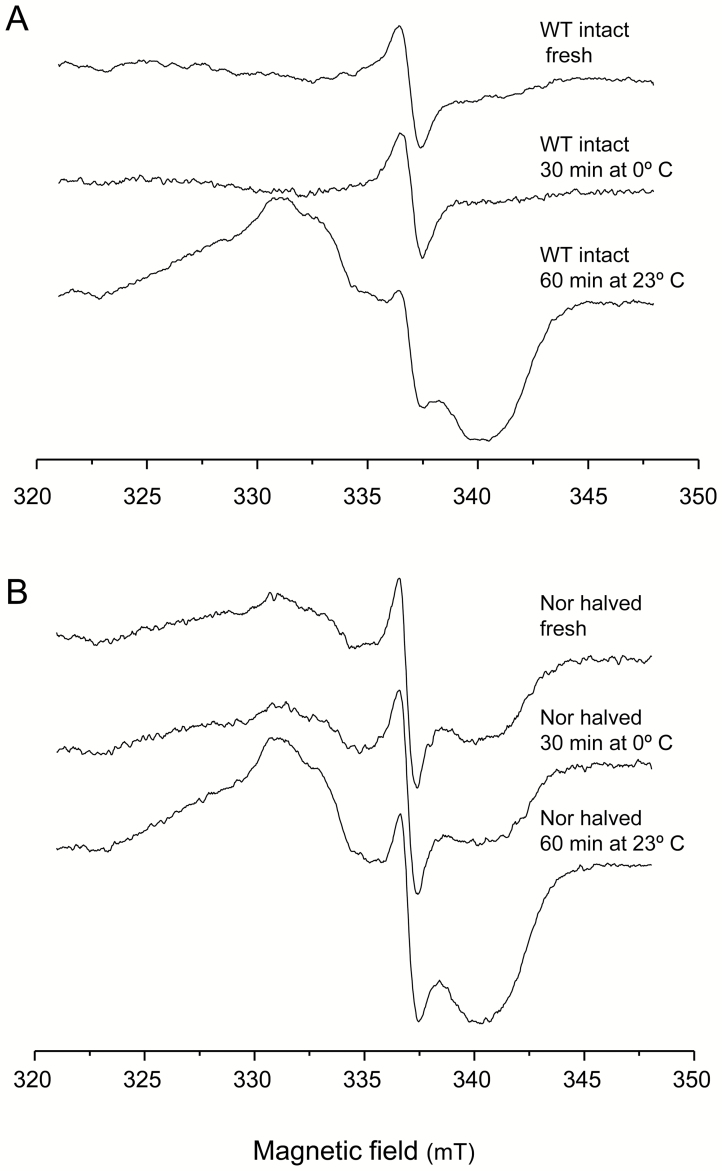
EPR spectra of soybean nodules showing artifactual production of Lb^2+^NO. Plants nodulated with the (A) WT and (B) *norC* strains were treated with 10 mM KNO_3_ for 3 d or 6 d, repectively. For (A), nodules were flash-frozen in liquid nitrogen immediately after detachment (fresh) or left to stand at 0 °C for 30 min or at 23 °C for 60 min. For (B), nodules were halved and immediately frozen in liquid nitrogen or left to stand at 0 °C for 30 min or at 23 °C for 60 min.

To verify the spurious production of Lb^2+^NO, we examined intact WT nodules that had been treated with NO_3_^−^ for 3 d. These nodules, when immediately analyzed or kept at 0 °C for 30 min, did not contain Lb^2+^NO, but this was produced if nodules were left at 23 °C for 60 min (Fig. 6A). In a parallel experiment, *norC* nodules that had been exposed to NO_3_^−^ for 6 d were cut in half. The nodules were then either immediately analyzed or left to stand at 0 °C for 30 min or at 23 °C for 60 min. The Lb^2+^NO content was similar in halved nodules immediately frozen in liquid nitrogen and in those kept on ice, but it increased after incubation at 23 °C (Fig. 6B). The temperature-sensitive production of NO in the infected zone is attributable to the denitrification enzymes, whose activities are induced by the decrease in O_2_ concentration after detaching the nodules (Arrese-Igor *et al.*, 1998; Bueno *et al.*, 2012).

Notably, our results are at odds with a previous EPR analysis of intact soybean nodules (Mathieu *et al.*, 1998). A detailed comparison of the spectra presented in the two studies points out some differences: (i) these authors assigned a magnetic field signal at *g*=3.3 to metal ions, mainly Fe^3+^ species, but we identified the signal of non-heme Fe^3+^ at *g*=4.3; (ii) they did not find any Mn^2+^ signal; and (iii) the spectrum of their preparation of Lb^2+^NO is not identical to our spectra of soybean or bean Lb^2+^NO. We obtained identical EPR spectra for Lb^2+^NO synthesized by two methods (Fig. 1), and the EPR signals of Lb^2+^NO combined numerically with the signals of intact nodules yielded the spectra that would be expected for nodules containing even small amounts of Lb^2+^NO (Fig. 3). We found exactly those spectra in soybean nodules deficient in NirK or Nor, indicating that, in our hands, EPR was a sensitive and specific method for Lb^2+^NO detection. However, we did not detect Lb^2+^NO in WT nodules with or without NO_3_^−^, in contrast to Mathieu *et al.* (1998). We cannot offer a conclusive explanation for all these discrepancies in the spectra and results, but they could be attributed to variations in measurement conditions, sample processing, the physiological state of nodules, or a combination of those factors.

### NO is detected with a fluorescent dye in the infected zone of nodules not treated with nitrate and in the parenchyma of soybean nodules

**Fig. 7.**
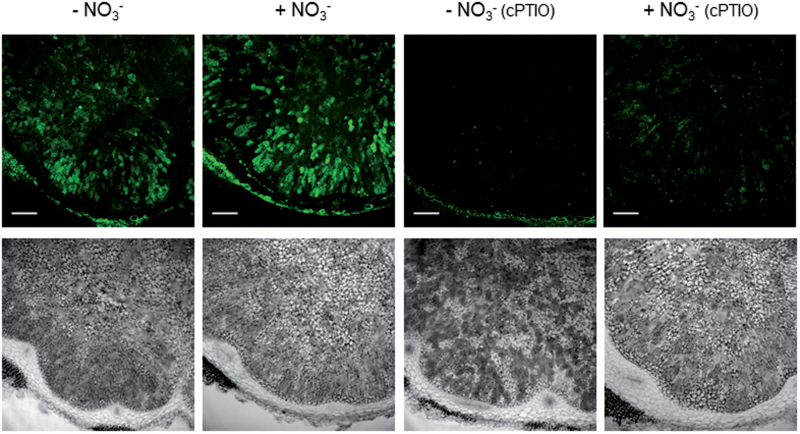
CLSM images showing NO localization in bean nodules. Plants were treated or not with 10 mM KNO_3_ for 4 d, but all of them were 30 d old when nodules were collected. Nodule sections were examined with identical settings. Lower panels are the bright-field images of the upper panels. Note the green fluorescence marking the presence of NO in the infected zone and its disappearance in nodule sections incubated with cPTIO. Scale bars=200 μm.

The results of EPR spectroscopy were tested using DAF-2 DA. This dye is deacetylated by intracellular esterases to DAF-2, which then reacts with NO under aerobic conditions to yield triazolofluorescein, a derivative that emits an intense green fluorescence. This method requires the use of strict controls to prove the absence of endogenous fluorescence in the plant tissue and the inhibition of the signal by the NO scavenger cPTIO (Mur *et al.*, 2011). We sectioned fresh bean and soybean nodules and immediately incubated the sections with the dye to allow its reaction with NO. For both types of nodules we ran parallel controls omitting the dye, which showed no background fluorescence. In bean nodules, the green fluorescence was localized in the infected zone of nodules not given NO_3_^−^ and was slightly enhanced in nodules treated with NO_3_^−^ (Fig. 7). The fluorescence signal was abolished upon incubation with cPTIO, indicating that it is genuinely due to NO and further confirming the absence of endogenous fluorescence.

**Fig. 8.**
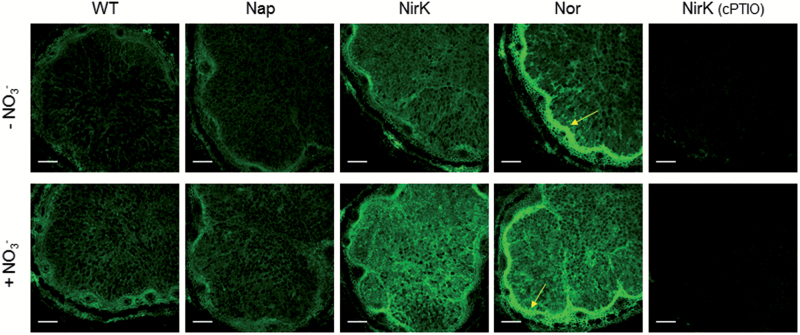
CLSM images showing NO localization in soybean nodules. Plants nodulated with the WT strain or with mutants defective in the enzymes Nap, NirK, or Nor were treated or not with 10 mM KNO_3_ for 6 d. All plants were 35 d old when nodules were collected. Nodule sections were examined with identical settings. Note the green fluorescence marking the presence of NO in the infected zone and in the nodule parenchyma (yellow arrows). The inhibition of the NO signal by cPTIO occurred in all nodule samples and the images of nodules lacking NirK are shown as an example. Scale bars=200 μm.

The fluorescence associated with NO was seen also in soybean nodules not treated with NO_3_^−^, but in this case the signal was localized both in the nodule parenchyma and in the infected zone (Fig. 8). The fluorescence intensity was similar in soybean nodules formed by the WT and *napA* strains, moderately higher in the infected zone of nodules of the *nirK* mutant, and even more intense in both regions of nodules of the *norC* mutant, especially in the parenchyma. Regarding the plants treated with NO_3_^−^ for 6 d, the nodules formed by the WT or *napA* strains displayed similar signal intensity, comparable with nodules not given NO_3_^−^ (Fig. 8). In contrast, the fluorescence signal increased with NO_3_^−^ in nodules of the *nirK* and *norC* mutants, and was particularly conspicuous in the infected zone (Fig. 8). As occurred with bean nodules, the fluorescence was suppressed by incubation of soybean nodule sections with cPTIO, and this observation also confirmed the absence of background signal (Fig. 8). Notably, the indeterminate nodules of *M. truncatula* formed by an *S. meliloti norB* mutant appeared to have enhanced NO levels but results are difficult to compare due to the major differences in growth patterns between the two types of nodules and also because details of NO_3_^−^ nutrition were not given (Meilhoc *et al.*, 2013).

The observation of an intense NO production in the soybean nodule parenchyma is noteworthy (Fig. 8). This region contains high concentrations of antioxidants (Dalton *et al.*, 1998) and co-localizes with the ODB that regulates the O_2_ flux into the infected zone (Minchin *et al.*, 2008). Because the electron transport chain of nodule mitochondria can generate NO (Horchani *et al.*, 2011), it is tempting to speculate that the observed increase in NO originates in the mitochondria as a result of the rapid O_2_ consumption in the nodule parenchyma linked to the operation of the ODB (Dalton *et al.*, 1998). Surprisingly, we could not detect a comparable NO signal in bean nodules under any of the examined conditions (Fig. 7), suggesting metabolic differences between these two determinate nodules.

### Inhibitor studies support a small contribution of plant nitrate reductase, but not of a nitric oxide synthase, activity to NO production in nodules

To gain information about the source of NO in nodules, we tested several known inhibitors of plant NR and animal NO synthase-like activity. Incubation of sections of NO_3_^−^-treated bean or soybean nodules with Na_2_WO_4_, an inhibitor of plant NR (Harper and Nicholas, 1978), slightly decreased the fluorescence intensity (Supplementary Fig. S6), indicating that this enzyme plays a secondary role in NO production compared with bacteroid denitrification. This conclusion is supported by the finding that the supply of NO_3_^−^ had little impact on NO production in bean nodules (Fig. 7) or in soybean nodules formed by the WT or *napA* strains (Fig. 8).

**Fig. 9.**
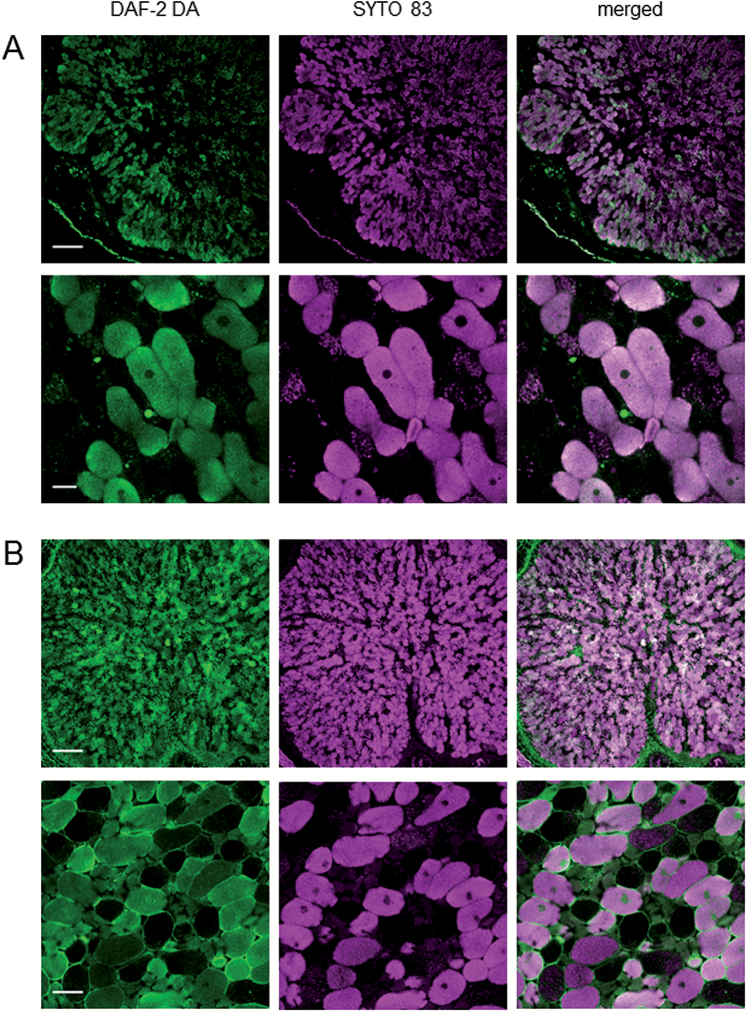
Co-localization of NO production (DAF-2 DA) and infected cells (SYTO 83) in (A) nodules of bean plants not treated with KNO_3_ and (B) nodules of soybean plants treated with KNO_3_ for 6 d. Similar results were obtained with bean nodules treated with KNO_3_ or with soybean nodules not treated with KNO_3_. SYTO 83 is a cell-permeant compound that exhibits intense fluorescence upon binding to nucleic acids. It intercalates in bacteroid DNA, marking the host infected cells. Scale bars=200 μm (A and B, upper panels); 25 μm (A, lower panels); 50 μm (B, lower panels).

In contrast, the incubation of nodule sections with l-NNA or l-NMMA, which are inhibitors of animal NO synthases, had no effect on the signal (Supplementary Fig. S6). Because some previous observations support the presence of NO synthase-like activity in nodules (Cueto *et al.*, 1996; Baudouin *et al.*, 2006), we tested these inhibitors at different concentrations and time exposures (1–5 mM, 1–2 h) but none of them decreased the fluorescence signal. This inconsistency could be due to metabolic differences between indeterminate and determinate nodules, and strongly suggest that pathways other than plant NR and NO synthase-like enzymes are operative in nodules. These pathways may be independent of NO_3_^−^ and especially operative in the infected zone. In bean nodules, the NO fluorescence signal was similarly intense without or with NO_3_^−^ (Fig. 7), and in both bean and soybean nodules a large part of the signal co-localized with the infected cells in the absence or presence of NO_3_^−^ (Fig. 9).

### EPR and fluorescence methods are complementary: EPR spectroscopy detects NO *in vivo* but is restricted to the infected zone, whereas fluorescent probes allow localization of the sites of potential NO production in nodule tissues

Our results of NO localization in bean and soybean nodules call into question a number of previous studies and reveal substantial discrepancies between EPR spectroscopy and fluorescent dyes. In the first place, the Lb^2+^NO observed in Lb preparations or nodule extracts (Maskall *et al.*, 1977; Kanayama *et al.*, 1990; Kanayama and Yamamoto, 1991) is artifactual because our EPR data show that Lb^2+^NO is absent from soybean nodules treated with NO_3_^−^ but is generated in intact nodules not immediately frozen as well as in nodule extracts. The exception may be the extracts from soybean nodules subjected to flooding and other hypoxic conditions because the corresponding intact nodules have a reasonably good EPR signal, which indicates genuine formation of Lb^2+^NO probably as a result of activation of denitrification enzymes (Meakin *et al.*, 2007; Sánchez *et al.*, 2010). In the second place, EPR spectroscopy shows that NO is produced in the infected zone of soybean nodules formed by the *nirK* and *norC* mutants, but the fluorometric method revealed that NO is also generated in bean and soybean nodules with or without NO_3_^−^, conditions in which Lb^2+^NO is not detected by EPR. The finding that NO formation is artifactually enhanced in nodules by sectioning or incubation at 23 °C (Fig. 6), which are unavoidable steps for the fluorometric detection of NO, casts doubts on the validity of this method in quantitative terms. The factors that may induce NO production during sample processing are the mechanical wounding caused by the detaching and slicing of nodules, the disruption of the microoxic conditions prevailing in the infected zone, the access of cortical NO_3_^−^ to the bacteroids, and the increase of denitrification enzyme activities in bacteroids at 23 °C. Additional potential pitfalls in the fluorometric detection of NO is that the DAF-2 DA probe reacts, at least *in vitro*, with oxidants such as peroxynitrite (Jourd’heuil, 2002) and antioxidants such as ascorbate and dehydroascorbate (Zhang *et al.*, 2002), modifying the fluorescence intensity attributed to NO. Therefore, fluorescent dyes should be used with essential controls (cPTIO) and only to assess the potential of the various nodule tissues to generate NO. However, EPR spectroscopy of intact nodules should be the method of choice to detect NO *in vivo*. Nevertheless, EPR is based on the detection of the Lb^2+^NO complex and hence has the drawback that it only allows the relative quantification of NO within the infected zone.

In conclusion, reliable EPR measurements of intact bean and soybean nodules treated or not with NO_3_^−^ show that Lb^2+^NO only accumulates in the infected zone of soybean nodules defective in NirK and Nor after 6 d with NO_3_^−^. These observations indicate that bacteroids are a major source of NO, that denitrification enzymes are required for NO homeostasis, and that Lb^2+^NO is not responsible for the inhibition of nitrogen fixation by NO_3_^−^. Our EPR data also reveal that Lb^2+^NO is artifactually generated if nodules are not examined immediately after detachment, and hence quantification of Lb^2+^NO in nodule extracts is not valid. On the other hand, fluorescent dyes with adequate controls are useful to localize relative NO production in the various nodule tissues. This method reveals NO formation in the infected zone of bean and soybean nodules and, interestingly, in the parenchyma of soybean nodules, suggesting a contribution of NO to the operation of the ODB. The NO-associated fluorescent signal was slightly decreased by inhibitors of NR but not of NO synthase, which is evidence of a rather minor contribution of plant NR to NO production in the presence of NO_3_^−^ and suggests the existence of NO_3_^−^- and arginine-independent pathways for NO production in nodules.

## Supplementary data

Supplementary data are available at *JXB* online.

Fig. S1. EPR spectra of purified soybean Lb*a* in different oxidation and ligand-binding states.

Fig. S2. EPR spectra of bean Lb*a*^3+^ and soybean Lb*a*^3+^ at different temperatures.

Fig. S3. EPR spectrum of intact bean nodules showing the signals corresponding to rhombic non-heme Fe^3+^, Mn^2+^, and organic radical species.

Fig. S4. EPR spectra of intact nodules from bean plants treated or not with 10 mM KNO_3_.

Fig. S5. EPR spectra of soluble extracts from bean and soybean nodules.

Fig. S6. Effect of enzyme inhibitors on NO production in soybean nodules.

Supplementary Figures

## Abbreviations

DAF-2 DA: 4,5-diaminofluorescein diacetate
Lb: leghemoglobin
Lb^2+^NO: nitrosyl-leghemoglobin
Nap: bacteroid periplasmic nitrate reductase
NirK: bacteroid respiratory nitrite reductase
NO: nitric oxide
Nor: bacteroid nitric oxide reductase
NOS: nitric oxide synthase
NiR: plant nitrite reductase
NR: plant nitrate reductase
ODB: oxygen diffusion barrier.
