# Supplementary material for: Redefining nitric oxide production in legume nodules through complementary insights from electron paramagnetic resonance spectroscopy and specific fluorescent probes
Source: J Exp Bot. 2018 Apr 26;69(15):3703–14. doi: 10.1093/jxb/ery159 (PMC6022593; doi:10.1093/jxb/ery159)
Supplement: Supplementary Figures [file ery159_suppl_supplementary_figures.pdf]

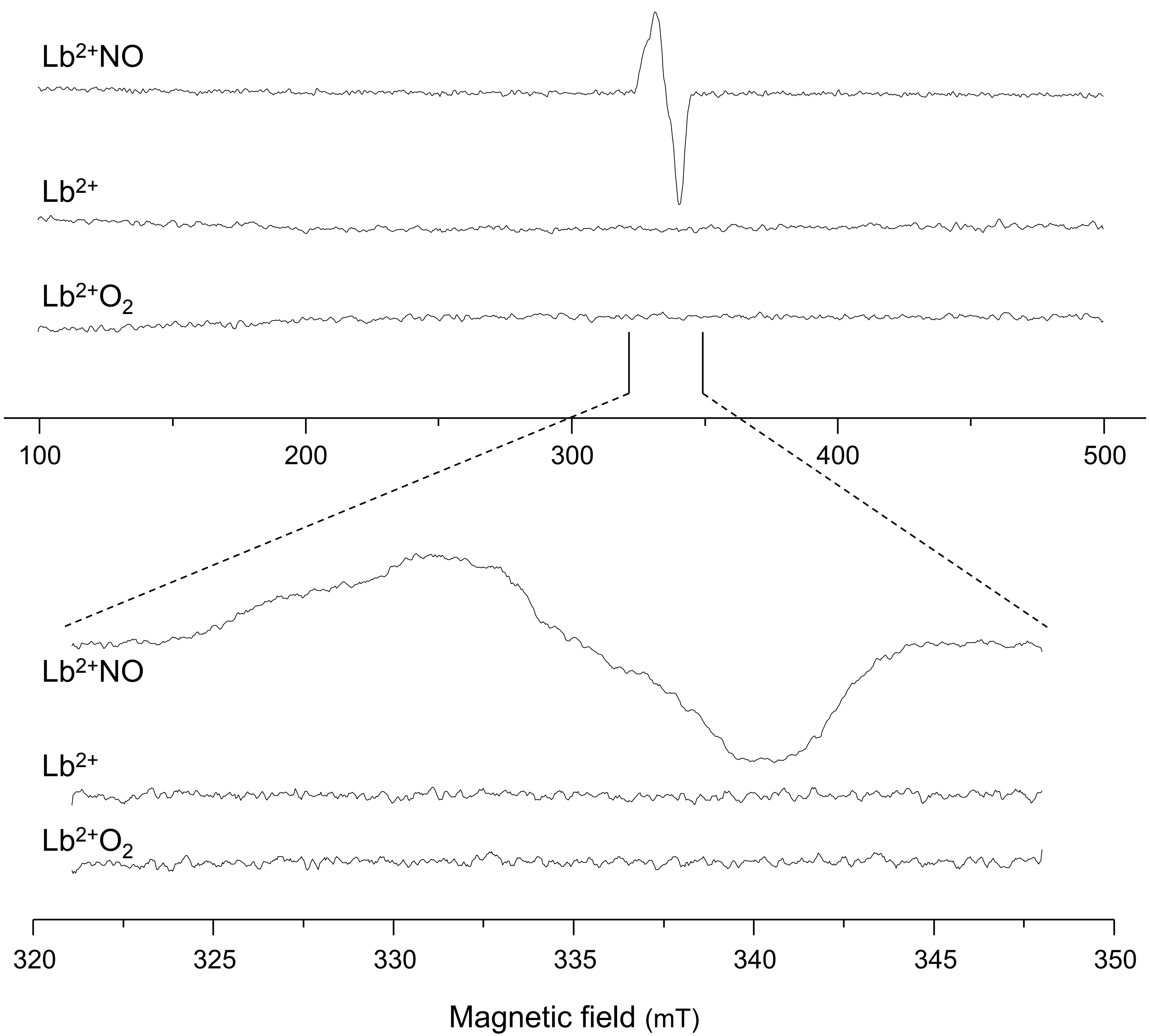

Figure S1. EPR spectra of purified soybean Lb*a* in different oxidation and ligand-binding states. The spectra are shown in two ranges of magnetic field.

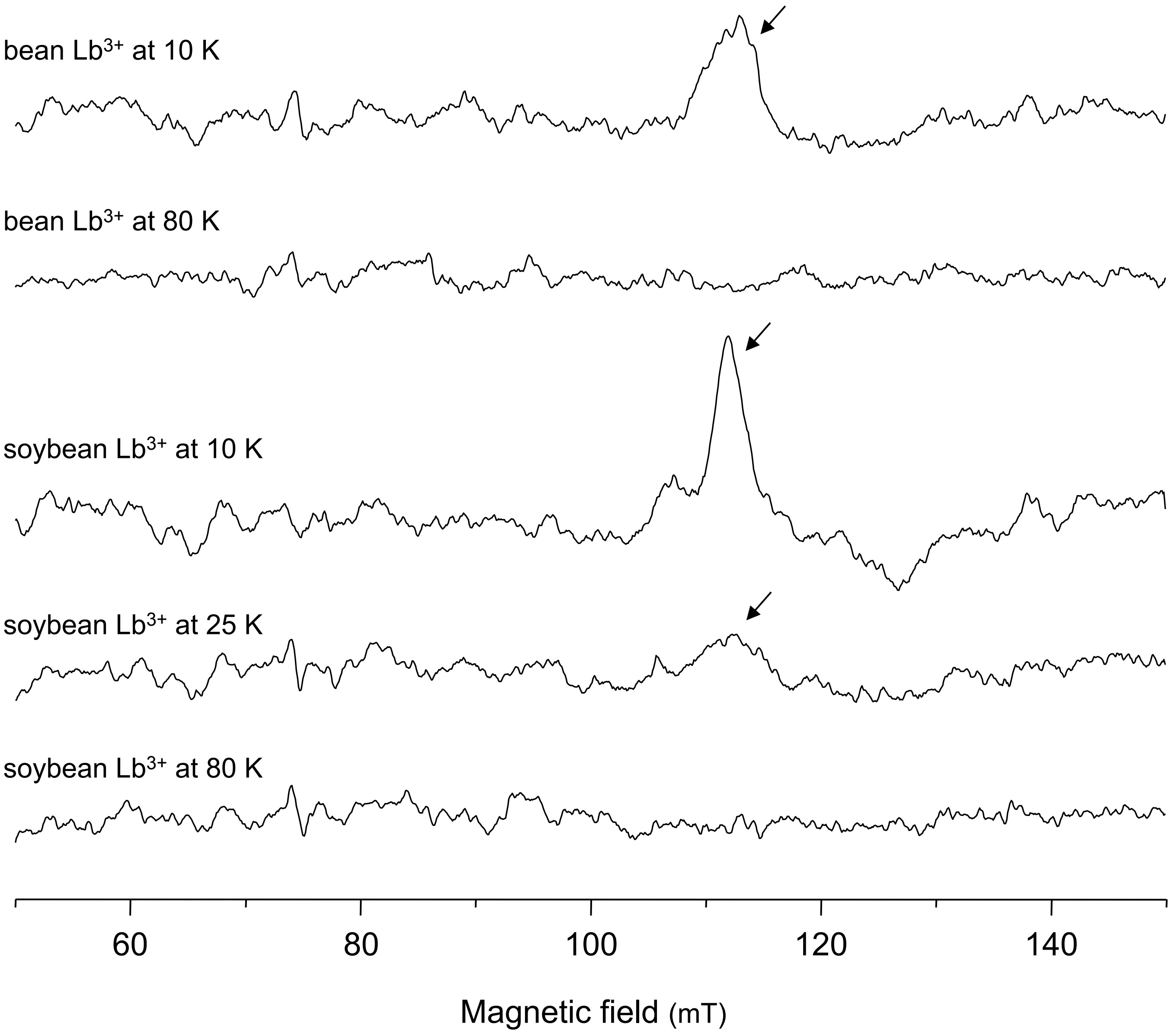

Figure S2. **EPR spectra of bean Lb*a*$^{3+}$ and soybean Lb*a*$^{3+}$ at different temperatures.** Arrows indicate the diagnostic peaks.

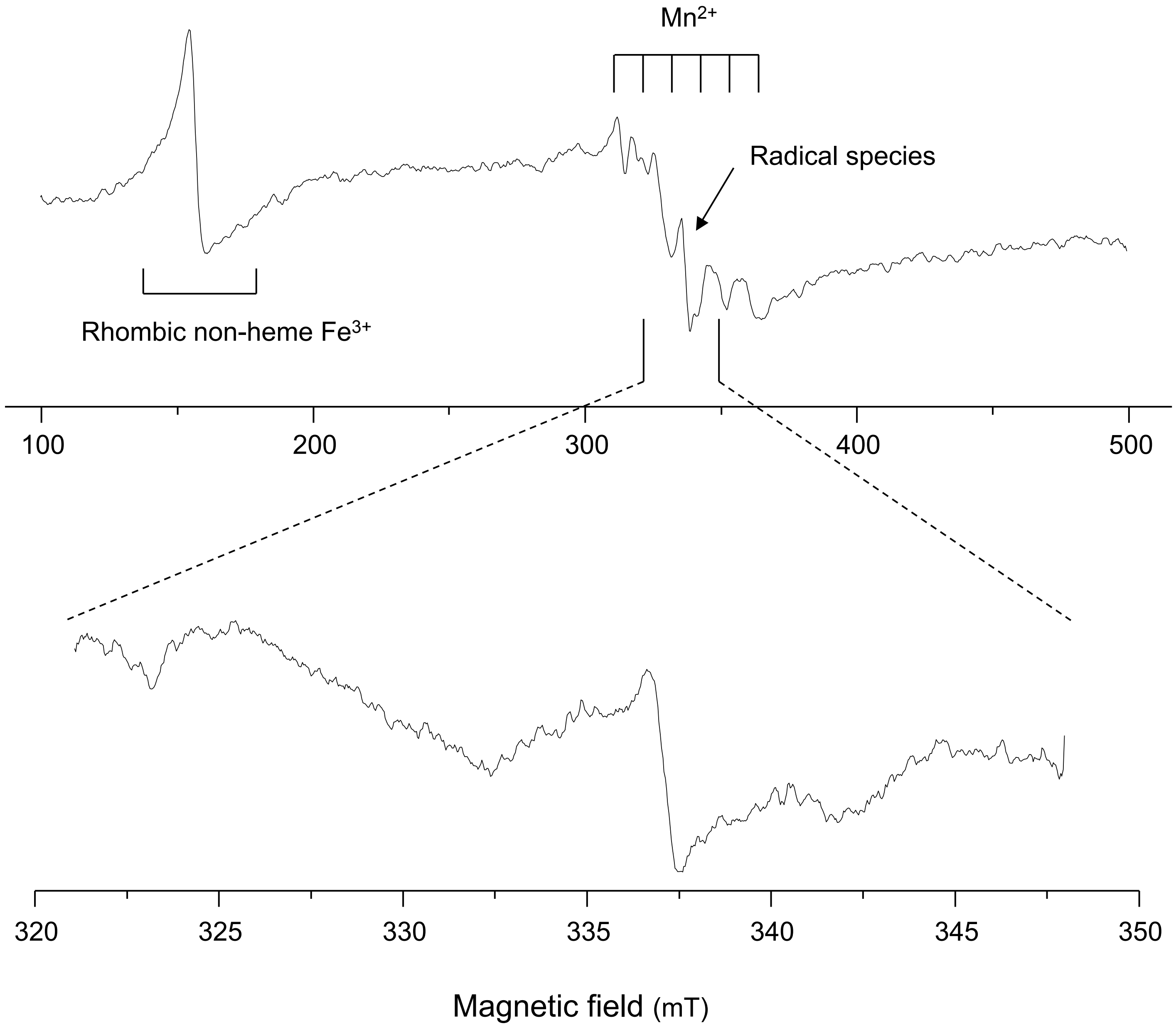

Figure S3. EPR spectrum of intact bean nodules showing the signals corresponding to rhombic non-heme Fe^{3+}, Mn^{2+}, and organic radical species.

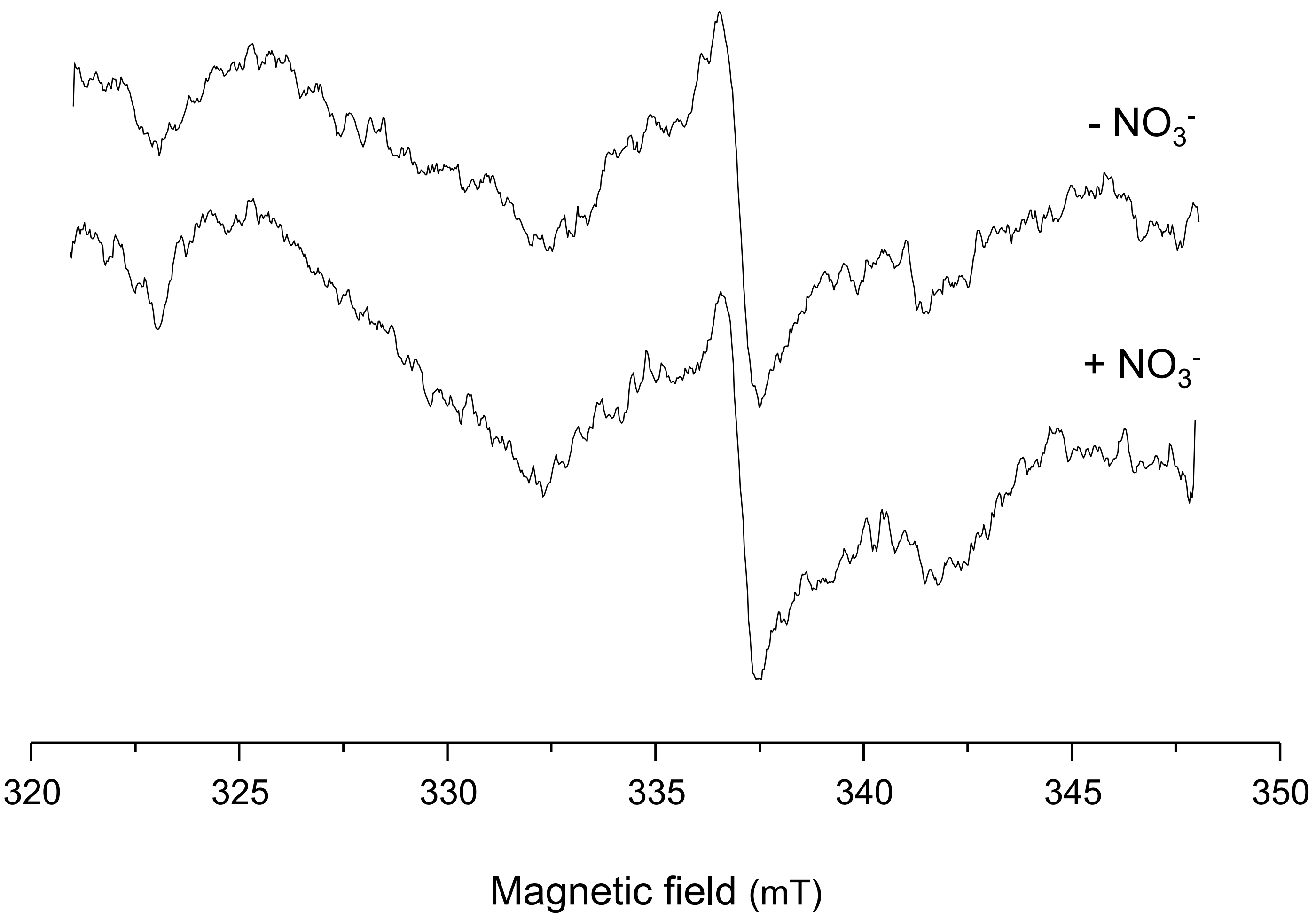

Figure S4. EPR spectra of intact nodules from bean plants treated or not with 10 mM KNO_3. Note that the $Lb^{2+}NO$ signal is absent and that the shoulders at 324 mT and 342 mT are associated with the Mn^{2+} signal (Supplementary Fig. S3).

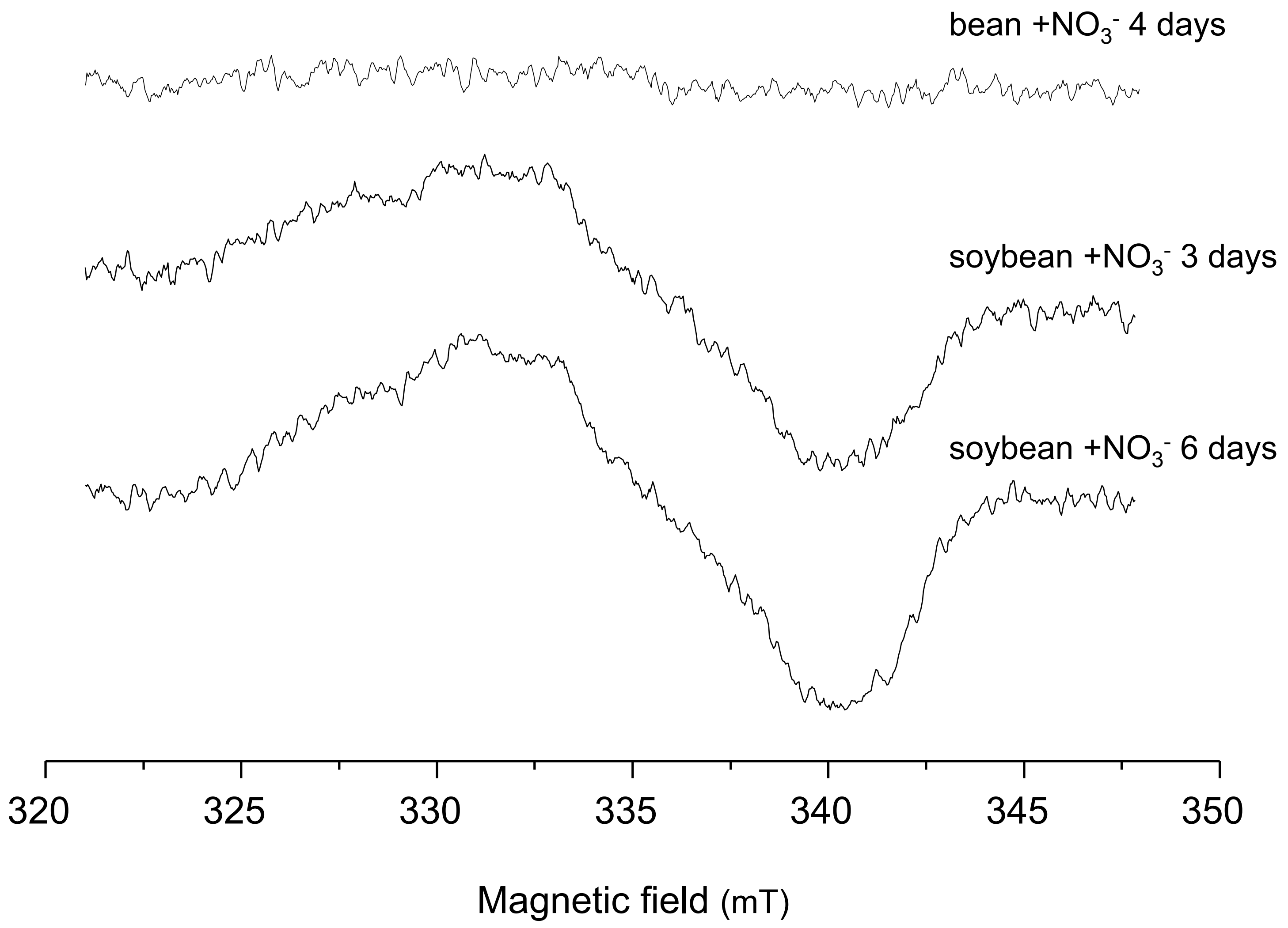

Figure S5. EPR spectra of soluble extracts from bean and soybean nodules. Plants were treated with 10 mM KNO_3 for the indicated times.

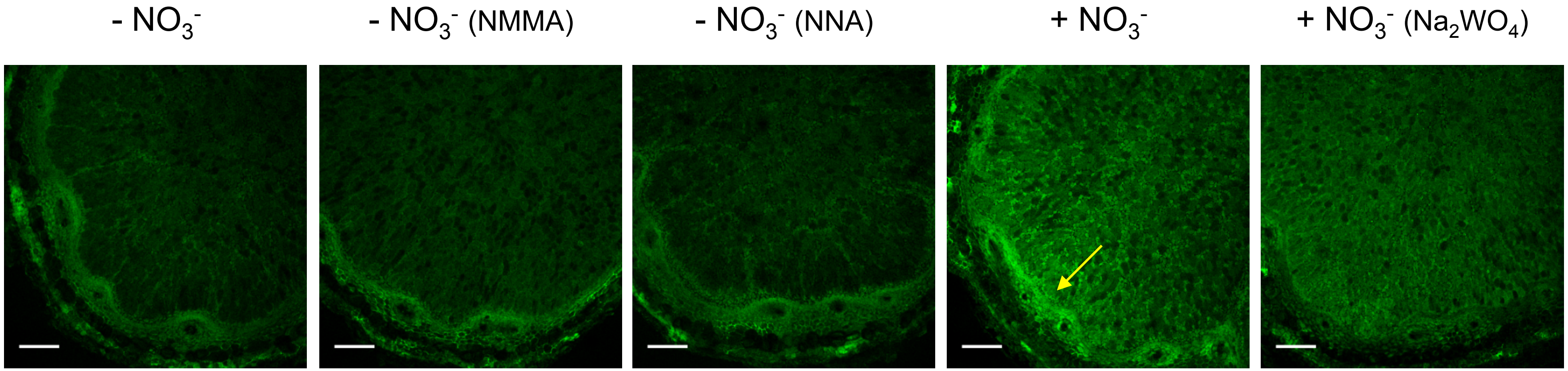

Figure S6. Effect of enzyme inhibitors on NO production in soybean nodules. Plants were treated or not with 10 mM KNO_3 for six days. Representative images of nodule sections incubated with the NO synthase inhibitors L-NMMA and L-NNA (1 mM , 1 h) or with the NR inhibitor Na_2WO_4 (5 mM, 2 h) are shown. Note the inhibition of NO signal with Na_2WO_4 in the infected zone and nodule parenchyma (arrow). Bars, 200 μm.
